# Assessing the repeatability of verbal autopsy for determining cause of death: two case studies among women of reproductive age in Burkina Faso and Indonesia

**DOI:** 10.1186/1478-7954-7-6

**Published:** 2009-05-05

**Authors:** Peter Byass, Lucia D'Ambruoso, Moctar Ouédraogo, S Nurul Qomariyah

**Affiliations:** 1 Immpact, University of Aberdeen, Aberdeen, AB25 2ZD, UK; 2 GREFSaD, Bobo-Dioulasso, Burkina Faso; 3 Immpact, Center for Family Welfare, Faculty of Public Health, University of Indonesia, Jakarta, Indonesia

## Abstract

**Background:**

Verbal autopsy (VA) is an established tool for assessing cause-specific mortality patterns in communities where deaths are not routinely medically certified, and is an important source of data on deaths among the poorer half of the world's population. However, the repeatability of the VA process has never been investigated, even though it is an important factor in its overall validity. This study analyses repeatability in terms of the overall VA process (from interview to cause-specific mortality fractions (CSMF)), as well as specifically for interview material and individual causes of death, using data from Burkina Faso and Indonesia.

**Methods:**

Two series of repeated VA interviews relating to women of reproductive age in Burkina Faso (n = 91) and Indonesia (n = 116) were analysed for repeatability in terms of interview material, individual causes of death and CSMFs. All the VA data were interpreted using the InterVA-M model, which provides 100% intrinsic repeatability for interpretation, and thus eliminated the need to consider variations or repeatability in physician coding.

**Results:**

The repeatability of the overall VA process from interview to CSMFs was good in both countries. Repeatability was moderate in the interview material, and lower in terms of individual causes of death. Burkinabé data were less repeatable than Indonesian, and repeatability also declined with longer recall periods between the death and interview, particularly after two years.

**Conclusion:**

While these analyses do not address the validity of the VA process in absolute terms, repeatability is a prerequisite for intrinsic validity. This study thus adds new understanding to the quest for reliable cause of death assessment in communities lacking routine medical certification of deaths, and confirms the status of VA as an important and reliable tool at the community level, but perhaps less so at the individual level.

## Background

Garenne and Faveau [1] recently set out a brief history of verbal enquiries into cause of death, including identifying some of the possible limitations, but without mentioning any aspect of repeatability of the process. Verbal autopsy (VA) has become an increasingly well-established approach for determining cause of death in populations lacking universal cause of death registration over the past two decades, and is a very important source of data on deaths among the poorer half of the world's people. Following early VA work in West Africa [2], there have been a number of efforts towards standardisation of VA procedures. Much of this has concentrated on the standardisation of interview questionnaires, culminating in WHO's recently published standards [3]. There has also been work on objective approaches to interpreting material from VA interviews [4-10], albeit with a possible trade-off between standardisation and subtlety of interpretation. Other studies have made comparisons between VA-derived cause of death and arguably "harder" evidence, such as hospital records for deaths occurring in institutions [9-13]. Some of these have been described as "validation" studies for VA, although they have generally only considered selected parts of the overall VA process. However, none of this work has objectively assessed the repeatability of the overall VA process (from individual interviews to aggregated mortality patterns), nor of its constituent parts (interviews, interpretations, individual causes of death, aggregated mortality patterns).

In this paper, we report the results of a study designed specifically to examine the repeatability of the VA process. The study was run in parallel in Burkina Faso and Indonesia, to allow comparison between two very different settings, both of which involved repeated VA interviews concerning deaths among women of reproductive age. We chose to exclude consideration of repeatability of the interpretation stage of the process, by using the InterVA-M model [14], which gives an intrinsic 100% repeatability, and also allows inter-country comparisons without needing to consider systematic differences between interpreters with different training and backgrounds.

Our major aim was to assess the repeatability of the overall VA process under operational conditions, from interview to aggregated mortality patterns, in the two different settings.

Subsidiary aims were:

1. to assess the repeatability of the interview stage of the VA process (in terms of material gathered in VA interviews);

2. to assess the repeatability of cause of death determination at the individual level;

3. to assess the repeatability of cause-specific mortality fractions (CSMF) determined at the population level.

It should be noted that this study did not aim to arrive at conclusions, other than on repeatability, about cause of death patterns in either setting, and not explain differences in mortality patterns between the two countries, evaluate the clinical validity of VA, nor draw conclusions for health planning.

## Methods

**Figure 1:**
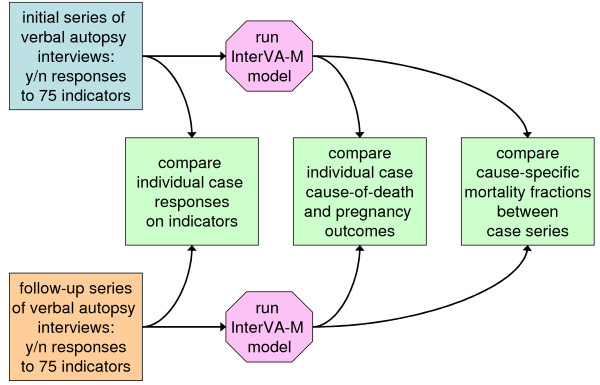
**Conceptual framework for assessing the repeatability of verbal autopsy interviews**.

In both Burkina Faso and Indonesia, large scale community-based surveys of mortality among women of reproductive age, with a particular focus on pregnancy-related mortality, were undertaken in 2005–6 (February to May 2006 in Burkina Faso and December 2005 to June 2006 in Indonesia) [15,16]. These surveys involved identifying deaths that had occurred among women of reproductive age and then undertaking verbal autopsy interviews. These interviews were structured to include the collection of the "indicators" (a range of 75 questions with "yes" or "no/unknown" responses, covering background, pregnancy status, clinical history, signs and symptoms before death and obstetric history) that are needed as the input material for the InterVA-M model [14]. This material from the interviews was interpreted using the InterVA-M model. For the purpose of these investigations into the repeatability of the VA process, subsamples of the originally identified cases were reselected, on a purposeful basis that approximately reflected the overall mortality patterns in the original surveys and were logistically feasible for re-interviewing. The majority of these cases either had no contact with health services around the time of death or case-notes were unavailable. These cases were then revisited in November 2007 (Burkina Faso) and January 2008 (Indonesia) and the VA interviews repeated, generally by different interviewers. Aspects of repeatability within the entire VA process, in both Burkina Faso and Indonesia, have been assessed within the conceptual framework shown in Figure 1.

The interview material thus consisted of individual sets of responses to the 75 possible InterVA-M indicators, and the repeatability of the interview stage of the overall process was assessed by calculating kappa statistics for each indicator, together with p-values assessing whether agreement between the original and follow-up interviews was significantly greater than that expected by chance.

The InterVA-M model was then run on all the original and follow-up interview material from both countries, to interpret likely pregnancy status and cause-of-death outcomes. The InterVA-M model generated, for each case, the most likely pregnancy status at the time of death (pregnant, delivered within 6 weeks or not pregnant within 6 weeks of death) with an associated likelihood. Then up to three likely alternative causes of death were generated, each with an associated likelihood. These likelihoods were used to ascribe fractional causes of death, as described previously [9]. As the model provides 100% repeatability between indicator input and pregnancy status or cause-of-death output, its performance was not part of this repeatability assessment. The individual level pregnancy status and cause of death likelihoods were summed over all individuals and the proportions calculated for each status/cause according to whether the same status or cause was or was not represented in the output from both an individual's original and follow-up interviews, within each country.

The individual pregnancy status and cause of death outputs were then combined into overall pregnancy status fractions and CSMFs for the population samples in Burkina Faso and Indonesia respectively, which were then compared in terms of magnitude and rank order.

Ethical approvals for the verbal autopsy studies in Burkina Faso and Indonesia, including follow-up interviews where needed, were granted by the Ministry of Health National Health Research Ethics Committee (Ouagadougou, Burkina Faso) and Centre MURAZ Institutional Review Board (Bobo-Dioulasso, Burkina Faso); by the Faculty of Public Health Research Ethics Committee at the University of Indonesia (Jakarta, Indonesia).

## Results

A total of 207 VA interviews were successfully repeated, 91 in Burkina Faso and 116 in Indonesia. The basic characteristics of these cases (both the deceased women and the interview respondents) are summarised in Table 1. Educational levels among both the deceased women and the VA respondents were much lower in Burkina Faso than in Indonesia, as were the availability of amenities such as piped water and television. Respondents in Indonesia were generally younger and more likely to be female than those in Burkina Faso. In Indonesia, in 86/116 interviews (74%), the same respondent was re-interviewed at follow-up. In Burkina Faso, the identities of the original respondents were not known and so comparison was not possible. The mean recall period from the death to the original interview was 26 months in Burkina Faso and 8 months in Indonesia, and similarly to the follow-up interview 46 months and 29 months respectively.

**Table 1:** Background characteristics for verbal autopsy interviews in Burkina Faso (n = 91) and Indonesia (n = 116).

**subject**	**characteristic**	**level**	**%**	
			
			Burkina Faso	Indonesia
deceased woman	education	primary	2.2	62.9
		
		secondary/higher	0	29.3
	
	work for income	yes	45.1	37.9
	
	place of death	health facility	51.7	45.7
	
	water supply	piped	1.1	30.2
	
	television	yes	2.2	50.0

respondent	sex	female	26.4	57.8
	
	age	under 40 yrs	30.8	44.0
	
	education	primary	8.8	62.1
		
		secondary/higher	2.2	11.3
	
	work for income	yes	90.1	62.1

Selected factors recorded at follow-up interviews, for women who had died and primary verbal autopsy interview respondents, are shown.

**Figure 2:**
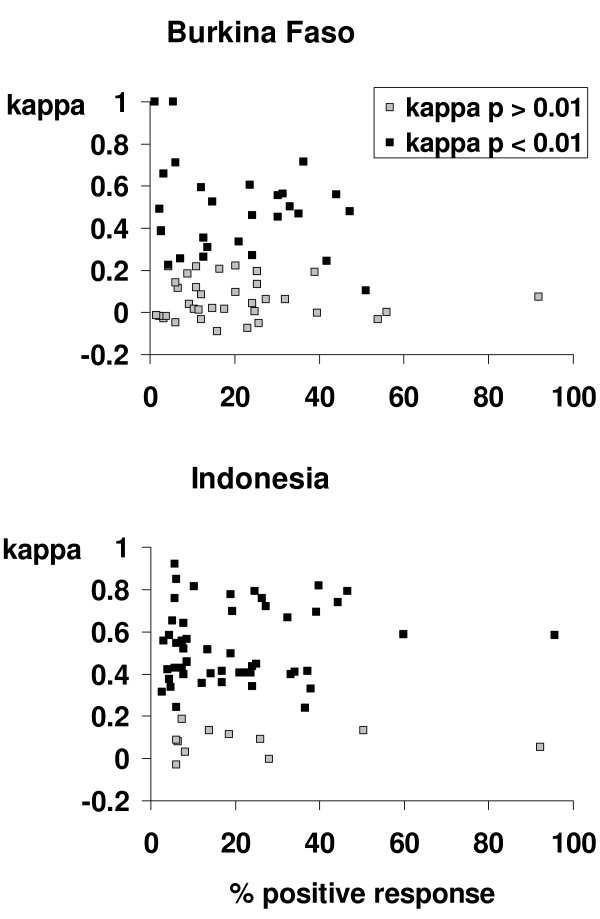
**Distribution of kappa statistics versus positive response rate for indicators obtained from repeated verbal autopsy interviews in Burkina Faso (n = 91) and Indonesia (n = 116)**.

Kappa statistics were calculated for each InterVA-M indicator in each country to assess the repeatability of the verbal autopsy interview process. The distribution of kappa statistics for all measurable InterVA-M indicators in relation to their positive response rate in each country are shown in Figure 2. Although the InterVA-M model captures a total of 75 indicators, some were either locally non-applicable or received no positive responses in one of the countries. Thus repeatability was measurable for 68 indicators in Burkina Faso and 63 in Indonesia. The mean κ was 0.24 (range -0.09 to 1.00) for Burkina Faso and 0.45 (range -0.03 to 0.92) for Indonesia. In Burkina Faso 30 out of 68 measurable indicators (44%) showed repeatability better than expected by chance at the p < 0.01 level. Full details are shown in Additional file 1. In Indonesia 52/63 indicators (83%) showed repeatability better than expected by chance at the p < 0.01 level. Full details are shown in Additional file 2.

Since recall period is obviously a potentially important factor in the repeatability of VA interviews, mean kappa statistics were also calculated separately for original interviews falling before or after the median recall time, for each country. In Burkina Faso the median time to first interview was 26 months, and for 51 indicators represented in interviews on both sides of the median, the mean κ was 0.27 for interviews up to and including the median, compared with 0.17 for interviews after the median. Of the 51 indicators, 76% had a lower κ value for interviews beyond the median recall time. In Indonesia, the median time to first interview was 7 months, and for 60 indicators the mean κ was 0.44 for interviews up to and including the median, compared with 0.45 for interviews after the median time.

Table 2 shows individual level agreement on pregnancy status and likely cause(s) of death for the Burkina Faso and Indonesian cases, after interpreting the VA interview material via the InterVA-M model. In Burkina Faso, 18.0% of the cause of death output was concordant between the original and follow-up interviews, while in Indonesia 25.0% was concordant. For pregnancy status, 67.2% and 85.0% respectively were concordant at the individual level.

**Table 2:** Individual repeatability of pregnancy status and cause-of-death assessment from repeated verbal autopsy interviews in Burkina Faso (n = 91) and Indonesia (n = 116)

	**Burkina Faso**		**Indonesia**	
	
**Likely cause of death**	% of interviews leading to this likely cause/status	of which, % with cause/status found in both interviews	% of interviews leading to this likely cause/status	of which, % with cause/status found in both interviews
Abortion related death	2.7	5.8	1.6	10.8

Anaemia	5.2	0	5.0	14.9

Cancer	2.9	20.3	4.1	20.5

Cardiovascular disease	5.7	5.0	6.3	24.8

Diabetes	4.1	19.0	4.9	35.2

Ectopic pregnancy	0.8	0	2.3	10.4

HIV/AIDS related death	5.0	9.9	1.0	0

Haemorrhage	8.8	17.2	14.4	28.4

Indeterminate	1.2	100.0	2.8	100.0

Kidney disease	2.0	13.6	3.2	18.7

Liver disease	1.9	0	4.2	23.3

Malaria	24.1	24.1	8.3	30.2

Obstructed labour	2.1	6.6	2.3	12.1

Other maternal cause	0.8	0	2.8	23.9

Pregnancy-induced hypertension	3.5	0	6.0	11.5

Pregnancy-related infection	20.8	29.5	17.8	22.1

Respiratory disease	0.6	0	0.2	0

Ruptured uterus	1.1	0	1.3	0

Suicide	0.9	0	0.7	35.3

Tuberculosis (pulmonary)	5.7	10.5	10.8	27.0

OVERALL	100.0	18.0	100.0	25.0

**Pregnancy status**				

not pregnant within 6 wks of death	6.7	66.0	8.0	82.2

pregnant within 6 wks of death	70.8	78.0	51.5	79.1

pregnant at death	22.7	34.0	40.5	90.1

OVERALL	100.0	67.2	100.0	85.0

Table 3 shows aggregated mortality as cause-specific mortality fractions (CSMF), together with ranked causes of death, in Burkina Faso and Indonesia, from the original and follow-up surveys. These results are presented at the population level, for each of the four series of VA interviews. Applying the Wilcoxon signed-rank test to the results from Burkina Faso and Indonesia gave results of z = 0.52 and 0.50 respectively, p > 0.6 in both cases. Thus there was no evidence of significant differences between original and follow-up CSMF patterns.

**Table 3:** Overall cause-specific mortality fractions and ranked causes, together with pregnancy status assessments, from verbal autopsies in Burkina Faso (n = 91) and Indonesia (n = 116), in original and follow-up interviews

	**Burkina Faso**				**Indonesia**			
	
	original interview		follow-up interview		original interview		follow-up interview	
**cause of death**	%	rank	%	rank	%	rank	%	rank

Abortion related death	3.6	10	1.8	11	1.3	17	1.9	15

Anaemia	6.8	5	3.6	7	5.4	7	4.7	8

Cancer	2.2	13	3.6	7	2.9	11	5.2	7

Cardiovascular disease	8.4	3	3.0	9	7.0	6	5.5	5

Diabetes	4.1	9	4.1	5	4.5	9	5.3	6

Ectopic pregnancy	0.3	19	1.4	14	1.8	15	2.9	13

HIV/AIDS related death	5.4	7	4.6	5	0.3	20	1.8	16

Haemorrhage	8.2	4	9.4	3	17.2	1	11.5	2

Indeterminate	1.2	17	0	18	2.8	12	0.9	18

Kidney disease	2.4	12	1.5	13	3.3	10	3.0	12

Liver disease	1.6	15	2.1	10	5.2	8	3.3	11

Malaria	19.6	1	30.0	1	8.8	4	7.8	4

Non-pregnancy related infection	0	20	0	18	0	21	0.9	18

Obstructed labour	2.6	11	1.7	12	2.6	13	2.0	14

Other maternal cause	1.6	16	0	18	2.1	14	3.6	10

Pregnancy-induced hypertension	5.8	6	1.2	15	8.3	5	3.7	9

Pregnancy-related infection	18.5	2	23.2	2	11.8	2	24.2	1

Respiratory disease	0	20	1.2	17	0.3	19	0	21

Ruptured uterus	2.2	14	0	18	1.6	16	1.0	17

Suicide	0.6	18	1.2	15	1.0	18	0.4	20

Tuberculosis (pulmonary)	5.0	8	6.4	4	11.8	3	10.6	3

**Pregnancy status**								

not pregnant within 6 wks of death	6.8	3	21.5	2	8.2	3	7.8	3

pregnant within 6 wks of death	70.9	1	67.0	1	51.4	1	51.6	1

pregnant at death	22.3	2	11.5	3	40.4	2	40.6	2

Table 4 shows the top five ranking causes of death and associated CSMFs from the original and follow-up surveys in both Burkina Faso and Indonesia, at the population level. In all four surveys, the top five causes of death accounted for approximately 60% of overall mortality.

**Table 4:** Top five causes of death from original and follow-up interviews in series of verbal autopsies in Burkina Faso (n = 91) and Indonesia (n = 116).

	**Burkina Faso**				**Indonesia**			
	
	original interview		follow-up interview		original interview		follow-up interview	
**rank**	cause of death	CSMF %	cause of death	CSMF %	cause of death	CSMF %	cause of death	CSMF %

1	malaria	19.6	Malaria	30.0	haemorrhage	17.2	pregnancy-related infection	24.2

2	pregnancy-related infection	18.5	pregnancy-related infection	23.2	pregnancy-related infection	11.8	haemorrhage	11.5

3	cardio-vascular disease	8.4	Haemorrhage	9.4	tuberculosis (pulmonary)	11.8	tuberculosis (pulmonary)	10.6

4	haemorrhage	8.2	tuberculosis (pulmonary)	6.4	malaria	8.8	malaria	7.8

5	anaemia	6.8	HIV/AIDS related death	4.6	pregnancy-induced hypertension	8.3	cardio-vascular disease	5.5

total		61.5		73.6		57.9		59.6

## Discussion

The concept of VA, from the interview, through interpretation, to population-level results, is a complex one. This study has investigated the repeatability of three different stages of this overall process, which is entirely novel, and capitalised on the use of the InterVA-M interpretative model in order to eliminate any subjective or inter-country variation in the process of interpreting the VA interview material.

### Methodological issues

It is difficult to discuss these findings extensively in relation to other work, since very little attention has previously been given to the repeatability of VA. All of our interviews were conducted in real field conditions, typical of settings in which VA is an important tool, and where deaths in hospital are rare. We happened to use material relating to women of reproductive age, although it is likely that similar findings would apply to other population groups. While it is possible that we may have influenced the conduct of the VA process by, exceptionally, undertaking follow-up interviews, we believe this is unlikely. In all cases the interval between the original and follow-up interviews was between one and two years, which probably minimised any effect associated with recalling the previous interview, even where the same respondent was involved. On the other hand, the additional accumulation of recall time since the death itself may have influenced the way in which original events were remembered, and thus reduced repeatability.

### VA respondents, recall and repeatability

In terms of the repeatability of the VA interview stage, it is clear that there were major differences between the interviews undertaken in Burkina Faso and Indonesia, with a markedly higher mean kappa statistic and proportion of indicators with non-chance agreement in Indonesia (Figure 2). We cannot say definitively what factors lie behind this major difference, although the marked differences observed in the characteristics of those who had died and of the interview respondents (Table 1) are putative factors. In particular, the lower educational levels, greater age and higher proportion of men among Burkinabé respondents could have resulted in a higher proportion of interviews in which the respondent did not clearly know or recall the sequence of events leading to death, with these shortcomings being reflected in inconsistencies between the original and follow-up interview material. However, the difference between the two countries was also confounded by different recall periods, and the analyses of kappa by recall time suggest that the effect of recall bias was much more pronounced in the longer recall periods experienced in Burkina Faso. From these findings it might be reasonable to conclude that VA interviews should, where possible, be undertaken within two years of death. However, since our objective here was to assess repeatability of VAs under operational conditions, rather than optimising the process, all these effects mean that our findings on reliability are possibly conservative.

When the individual-level pregnancy status and cause of death outputs were compared between the original and follow-up interviews, there was a disappointing lack of concordance, though this was perhaps not surprising given the extent of non-agreement between the interview material, particularly from Burkina Faso (Table 2). This finding supports the view that VA material may not be particularly well suited to individual-level cause of death determination [10], at least under the operational conditions encountered here.

However, when the output data were considered at the population level, there was a much clearer sense of agreement between the original and follow-up material (Table 3). As might be expected from the repeatability results at the first stage, there were still greater discrepancies in the Burkina Faso results compared with those from Indonesia. There were however, as would be expected, marked differences persisting in the overall patterns between the two countries, counteracting any suggestion that the whole VA process might amount to some kind of reduction to lowest common factors.

### Implications of VA repeatability for health policy

Taking a public health perspective, Table 4 considers repeatability of the VA process in the context of the often-asked question "what are the major causes of mortality?". It is clear that, for women both in Burkina Faso and Indonesia, there was very good repeatability between the original and follow-up VAs in terms of generating summary information for health policy and planning, even though the specific country findings were, as expected, different.

The relative levels of repeatability, both between the two country settings involved and between the different stages of the VA process, are interesting. The obvious differences between the two countries make clear that the whole VA process is context-dependent, since it might reasonably be inferred that in a context where repeatability is lower, then the intrinsic validity of one-time interview material would also be lower. The effects of non-repeatability at different stages of the VA process are also interesting – our results suggest that, given moderate repeatability within the original interviews, the repeatability of cause of death at the individual level is seriously compromised. However, when a reasonable group of individual VAs are taken as aggregate entities (around 100 per setting in these results) then our findings suggest that there is some recovery of repeatability, certainly to levels that appear to be acceptable in terms of generating aggregate data for health planning. The effects behind this are not easy to quantify, but it seems likely that some of the non-repeatability of details at the interview stage may have the effect of tipping the balance between possible causes at the individual level, but with many such differences then cancelling out on aggregating causes to the population level.

### Why repeatability?

Repeatability is often not explicitly assessed for health measurement tools – so why is it important in relation to VA and what do the results tell us? The origins of VA – as a proxy source of cause of death data in the absence of medical certification – has sometimes led to confusion about its fundamental nature. While it is not generally argued that VA should be used as a direct replacement for medical certification at the individual level, this principle has not always been made explicit in VA work. Consequently, attempts to validate VA [9-13] have tended to struggle for lack of clear, appropriate gold standards and methods, and the role of VA has to some extent been left in an uncertain position. At the same time, the rigour of medical certification of death is often not critically evaluated [17].

We therefore offer these analyses of VA repeatability as a fresh viewpoint on the overall process, to shed some light on the practice of VA under realistic operational conditions and the value of the ensuing results. This partly follows from our previous consideration of the question "Who needs cause of death data?" [18], since VA cannot be considered as reducible to a single one-size-fits-all tool, and must be contextualised.

At least in contexts emphasising community-level cause-specific mortality, the findings of these repeatability analyses are encouraging. Although good repeatability does not guarantee good validity, at least it suggests that intrinsic validity is not compromised by random effects in the overall process. It also became clear that the longer recall periods associated with some of the Burkinabé interviews were detrimental to repeatability – and so presumably to validity. At the same time, overall repeatability was lower in Burkina Faso than in Indonesia, possibly because of different respondent profiles, which again emphasises the importance of considering VA material contextually, rather than simply in terms of standardised methods.

## Conclusion

The overall process of VA, from interview to CSMF, has been shown to have good repeatability for two very different communities. However, VA outcomes were less repeatable at the individual level, and recall periods beyond two years compromised repeatability. Although repeatability does not demonstrate validity, it is a prerequisite, and so this study adds new understanding to the quest for reliable cause of death assessment in communities lacking routine medical certification of deaths.

## Competing interests

The authors declare that they have no competing interests.

## Authors' contributions

PB devised the study; all authors participated in field work; MO and SNQ coordinated work in Burkina Faso and Indonesia respectively and prepared datasets; PB & LD carried out analysis and drafting.

## Supplementary Material

Additional File 1 **Repeatability of VA indicators in Burkina Faso**. Details of repeatability for each verbal autopsy indicator from a series of 91 repeated interviews in Burkina Faso (by ascending κ values within each category). Click here for file

Additional File 2 **Repeatability of VA indicators in Indonesia**. Details of repeatability for each verbal autopsy indicator from a series of 116 repeated interviews in Burkina Faso (by ascending κ values within each category). Click here for file
