# Supplementary material for: Assessing the repeatability of verbal autopsy for determining cause of death: two case studies among women of reproductive age in Burkina Faso and Indonesia
Source: Popul Health Metr. 2009 May 5;7:6. doi: 10.1186/1478-7954-7-6 (PMC2679716; doi:10.1186/1478-7954-7-6)
Supplement: Additional File 1 — Repeatability of VA indicators in Burkina Faso. Details of repeatability for each verbal autopsy indicator from a series of 91 repeated interviews in Burkina Faso (by ascending κ values within each category). [file 1478-7954-7-6-S1.pdf]

category	indicator	% positive responses	% observed agreement	% expected agreement	κ	p for observed > expected
background	married	91.8	85.7	84.6	0.074	0.209
	20-34 yrs at death	51.1	63.7	49.7	0.104	0.004
	under 20 yrs at death	13.7	83.5	76.2	0.309	0.001
	died in wet season	30.2	76.9	57.7	0.455	0.000
	35+ years at death	35.2	75.8	54.4	0.470	0.000
pregnancy status	had ever been pregnant	56.0	14.3	14.0	0.003	0.348
	early pregnancy ended	6.6	89.0	87.6	0.115	0.121
	not pregnant at death	24.2	71.4	60.9	0.269	0.001
	pregnant at death	20.9	78.0	67.0	0.335	0.001
	breast feeding at death	24.2	80.2	63.3	0.461	0.000
	delivered within 6 wks	47.3	73.6	49.3	0.480	0.000
	first pregnancy	14.8	87.9	74.4	0.527	0.000
	> 4 pregnancies	44.0	78.0	50.1	0.559	0.000
clinical history	HIV/AIDS diagnosis	3.3	93.4	93.6	-0.030	0.620
	suspected suicide	1.6	96.7	96.8	-0.015	0.560
	recent injury	1.6	96.7	96.8	-0.015	0.560
	malaria diagnosis	17.6	71.4	71.0	0.015	0.443
	liver disease diagnosis	9.3	81.3	82.0	0.040	0.737
	CVD diagnosis	12.1	80.2	78.4	0.086	0.185
	had a previous C-sect	2.7	96.7	94.6	0.389	0.000
	TB diagnosis	2.2	97.8	95.7	0.491	0.000
	malignancy diagnosis	1.1	100.0	97.8	1.000	0.000
signs and symptoms before death	persistent fever	15.9	70.3	72.7	-0.088	0.815
	blurred vision	25.8	59.3	61.4	-0.053	0.698
	breathless	53.8	40.7	42.5	-0.032	0.661
	pallor or anaemia	39.6	51.7	51.8	-0.003	0.512
	acute fever	24.7	57.1	56.9	0.004	0.476
	swollen body	10.4	81.3	81.0	0.017	0.432
	abdominal pain	24.2	64.8	63.2	0.043	0.339
	collapse	31.9	53.9	56.5	0.061	0.719
	in bed during day	27.5	62.6	60.1	0.063	0.274
	jaundice	20.3	70.3	67.1	0.098	0.164
	fever with shivering	25.3	64.8	59.3	0.136	0.054
	night sweats	39.0	61.5	52.3	0.193	0.033
	recurrent fever	25.3	69.2	61.8	0.194	0.029
	coma >24h	16.5	78.0	72.3	0.208	0.021
	foul-smelling discharge	4.4	93.4	91.6	0.216	0.020
	stiff neck	11.0	84.6	80.3	0.217	0.017
	swollen feet	20.3	74.7	67.6	0.220	0.018
	swollen glands	4.4	93.4	91.5	0.224	0.006
	weight loss	41.8	62.6	50.5	0.245	0.008
	swollen face	12.6	83.5	77.6	0.263	0.004
	persistent cough	12.6	85.7	77.9	0.355	0.000

	coughing blood	2.7	96.7	94.7	0.384	0.000
	transfusion needed	30.2	81.3	57.8	0.557	0.000
	ever had fits	12.1	91.2	78.4	0.594	0.000
obstetric record	unwanted pregnancy	23.1	56.0	59.6	-0.074	0.882
	died in labour	6.0	87.9	88.5	-0.050	0.708
	abnormal baby position	12.1	78.0	78.7	-0.033	0.624
	assisted delivery	3.8	92.3	92.5	-0.019	0.605
	baby part prolapsed	2.2	95.6	95.7	-0.017	0.573
	hysterectomy	1.6	96.7	96.8	-0.015	0.560
	big baby	11.5	79.1	78.9	0.013	0.442
	fits during pregnancy	14.8	74.7	74.2	0.019	0.423
	BP raised in pregnancy	11.0	82.4	80.1	0.119	0.104
	first trimester death	6.0	90.1	88.5	0.141	0.063
	labour >24h	8.8	86.8	83.9	0.183	0.035
	bleeding at delivery	25.3	69.2	61.8	0.194	0.029
	bleeding in 1st trimester	7.1	90.1	86.7	0.255	0.007
	delivered at home	20.9	78.0	66.9	0.337	0.001
	attempted termination	2.7	96.7	94.6	0.389	0.000
	delivered in a facility	33.0	78.0	55.7	0.504	0.000
	professional care at del	31.3	81.3	56.9	0.563	0.000
	death within 24h of del	23.6	85.7	63.9	0.605	0.000
	multiple pregnancy	3.3	97.8	93.6	0.657	0.000
	placenta retained	6.0	96.7	88.6	0.710	0.000
	baby delivered alive	36.3	86.8	53.8	0.715	0.000
	delivered by C-section	5.5	100.0	89.6	1.000	0.000
