# Supplementary material for: Assessing the repeatability of verbal autopsy for determining cause of death: two case studies among women of reproductive age in Burkina Faso and Indonesia
Source: Popul Health Metr. 2009 May 5;7:6. doi: 10.1186/1478-7954-7-6 (PMC2679716; doi:10.1186/1478-7954-7-6)
Supplement: Additional File 2 — Repeatability of VA indicators in Indonesia. Details of repeatability for each verbal autopsy indicator from a series of 116 repeated interviews in Burkina Faso (by ascending κ values within each category). [file 1478-7954-7-6-S2.pdf]

category	indicator	% positive responses	% observed agreement	% expected agreement	κ	p for observed > expected
background	died in wet season	34.1	73.3	54.8	0.409	0.000
	under 20 yrs at death	7.3	94.0	86.4	0.556	0.000
	married	95.7	96.6	91.8	0.582	0.000
	20-34 yrs at death	59.9	80.2	52.0	0.587	0.000
	35+ years at death	32.3	85.3	56.2	0.665	0.000
pregnancy status	had ever been pregnant	92.2	86.2	85.0	0.055	0.067
	early pregnancy ended	3.9	95.7	92.5	0.422	0.000
	had a previous C-sect	5.2	96.6	90.1	0.651	0.000
	pregnant at death	39.2	85.3	52.2	0.693	0.000
	> 4 pregnancies	27.2	88.8	60.1	0.719	0.000
	first pregnancy	24.6	92.2	62.9	0.791	0.000
	delivered within 6 wks	46.6	89.7	50.1	0.793	0.000
	not pregnant at death	10.3	96.6	81.5	0.814	0.000
clinical history	recent injury	6.5	88.8	87.8	0.081	0.177
	recent surgery	2.6	96.6	95.0	0.316	0.000
	CVD diagnosis	4.7	94.0	90.9	0.339	0.000
	TB diagnosis	8.6	91.4	84.1	0.457	0.000
	liver disease diagnosis	6.0	94.8	88.6	0.545	0.000
signs and symptoms before death	stiff neck	6.0	87.9	88.3	-0.031	0.686
	foul-smelling discharge	8.2	85.3	84.9	0.031	0.362
	antibiotics needed	25.9	63.8	60.2	0.091	0.138
	collapse	18.5	73.3	69.8	0.116	0.105
	blurred vision	13.8	79.3	76.2	0.132	0.075
	pallor or anaemia	50.4	56.0	49.2	0.135	0.066
	persistent fever	7.3	88.8	86.2	0.186	0.013
	night sweats	36.6	64.7	53.5	0.239	0.005
	in bed during day	37.9	67.2	51.1	0.330	0.000
	abdominal pain	24.1	75.9	63.2	0.343	0.000
	coma >24h	12.1	86.2	78.5	0.357	0.000
	jaundice	16.8	81.9	71.7	0.360	0.000
	acute fever	33.2	73.3	55.7	0.398	0.000
	swollen body	7.8	91.4	85.7	0.398	0.000
	fever with shivering	21.1	80.2	66.7	0.405	0.000
	recurrent fever	22.4	79.3	65.2	0.405	0.000
	weight loss	37.1	72.4	53.0	0.413	0.000
	persistent cough	16.8	83.6	72.0	0.415	0.000
	swollen glands	5.6	94.0	89.4	0.430	0.000
	breathless	24.1	79.3	63.4	0.435	0.000
	swollen feet	25.0	79.3	62.5	0.449	0.000
	transfusion needed	19.0	84.5	69.3	0.495	0.000
	ever had fits	13.4	88.8	76.8	0.516	0.000
	coughing blood	7.8	93.1	85.7	0.519	0.000
	swollen face	7.8	94.8	85.7	0.639	0.000

obstetric record	unwanted pregnancy	28.0	59.5	59.6	-0.004	0.516
	bleeding in 1st trimester	6.0	89.7	88.6	0.089	0.166
	labour >24h	6.0	91.4	88.6	0.244	0.003
	died in labour	4.3	94.8	91.7	0.374	0.000
	big baby	7.8	91.4	85.7	0.398	0.000
	death within 24h of del	14.2	85.3	75.5	0.402	0.000
	bleeding at delivery	23.7	78.5	63.8	0.404	0.000
	fits during pregnancy	7.3	92.2	86.4	0.429	0.000
	placenta retained	8.6	91.4	84.2	0.455	0.000
	baby part prolapsed	3.0	97.4	94.1	0.558	0.000
	first trimester death	8.6	93.1	84.2	0.564	0.000
	abnormal baby position	4.3	96.6	91.7	0.585	0.000
	BP raised in pregnancy	19.4	90.5	68.7	0.697	0.000
	baby delivered alive	44.4	87.1	50.6	0.738	0.000
	professional care at del	26.3	90.5	61.2	0.756	0.000
	multiple pregnancy	5.6	97.4	89.4	0.756	0.000
	delivered in a facility	19.0	93.1	69.3	0.776	0.000
	delivered at home	39.7	91.4	52.1	0.820	0.000
	assisted delivery	6.0	98.3	88.7	0.848	0.000
	delivered by C-section	5.6	99.1	89.4	0.919	0.000
